# Steroid hormone profiling in obese and nonobese women with polycystic ovary syndrome

**DOI:** 10.1038/s41598-017-14534-2

**Published:** 2017-10-26

**Authors:** Yuying Deng, Yifei Zhang, Shengxian Li, Wenzhong Zhou, Lei Ye, Lihua Wang, Tao Tao, Junjie Gu, Zuwei Yang, Dandan Zhao, Weiqiong Gu, Jie Hong, Guang Ning, Wei Liu, Weiqing Wang

**Affiliations:** 1 Shanghai National Clinical Research Center for Endocrine and Metabolic Diseases, Key Laboratory for Endocrine and Metabolic Diseases of Chinese Ministry of Health, Shanghai Institute of Endocrine and Metabolic Diseases, Ruijin Hospital, Shanghai Jiaotong University School of Medicine, 197 Ruijin Er Road, Shanghai, 200025 China; 2 Department of Endocrinology, Ren Ji Hospital, Shanghai Jiao Tong University School of Medicine, 160 Pujian Road, Shanghai, 200127 China

## Abstract

The study explored differences in the steroidogenic pathway between obese and nonobese women with polycystic ovary syndrome (PCOS) using liquid chromatography-tandem mass spectrometry (LC-MS/MS). 1044 women with PCOS (including 350 lean, 312 overweight and 382 obese) and 366 control women without PCOS (including 203 lean, 32 overweight and 131 obese) were enrolled. The differences in steroid hormones were amplified in lean PCOS versus lean controls compared with obese PCOS versus obese controls. Compared with obese PCOS, lean PCOS demonstrated increased dehydroepiandrosterone sulfate (*P* = 0.015), 17-hydropregnenolone (*P* = 0.003), 17-hydroprogesterone (17-OHP) (*P* < 0.001), progesterone (*P* < 0.001) and estrone (*P* < 0.001) levels. Enzyme activity evaluation showed that lean PCOS had increased activity of P450c17 (17-hydropregnenolone/pregnenolone, *P* < 0.001), P450aro (*P* < 0.001), 3βHSD2 (progesterone/ pregnenolone and 17-OHP/17-hydropregnenolone, both *P* < 0.001) and decreased activity of P450c21(11-deoxycorticorsterone/progesterone and 11-deoxycortisol/17-OHP, *P* < 0.001). Moreover, we found higher frequencies of CYP21A2- (encoding P450c21) c.552 C > G (p. D184E) in lean PCOS compared with obese PCOS patients (*P* = 0.006). In conclusion, this study demonstrated for the first time that the adrenal-specific enzyme P450c21 showed decreased activity in lean PCOS patients, and that the adrenal androgen excess may play different roles in lean and obese PCOS patients, which represents as different enzyme activity in the steroidogenic pathway.

## Introduction

Polycystic ovary syndrome (PCOS) is a common endocrine disorder affecting 5.6–21.3% of women of reproductive age worldwide^1^, and is frequently associated with various metabolic risk factors^2^. The pathophysiology of PCOS varies and different factors interact with one another^3^. Hyperandrogenism is considered as a cardinal element in the pathogenesis of PCOS, while insulin resistance and its accompanying obesity play both direct and indirect roles in the pathogenesis of androgen excess in PCOS^4^. Although obesity affects 50–80% patients with PCOS^4,5^, normal-weight patients still account for a certain proportion of the population with PCOS. These normal-weight PCOS patients present less insulin resistance and fewer metabolic risk factors, which suggests that the pathogenesis of this group may differ from that of obese PCOS patients.

Although the ovaries have been reported to be the main source of androgen excess in PCOS, excess adrenal androgen levels and adrenal dysfunction have also been observed in PCOS patients^6^. Dehydroepiandrosterone sulfate (DHEAS), which has been reported to derive almost exclusively from the adrenal glands^7^, was found to be high in 22–25% of patients with PCOS^8^. A negative association between DHEAS and BMI or fasting insulin among PCOS patients has been reported by Kumar *et al*.^9^ and Moran *et al*.^10^, suggesting that the proportion of adrenal androgen excess may be higher in nonobese PCOS patients. However, these studies have mainly focused on the end-product of adrenal androgen synthesis, while the changes in the intermediate steroid hormones, and the activity of related enzymes in the steroidogenic pathway are incompletely understood. Furthermore, whether these findings in steroid hormone profiling lead to the understanding of the complicated pathophysiology in PCOS remains to be investigated.

Therefore, in the present study, we measured 14 steroid hormones in PCOS patients and controls, using liquid chromatography-tandem mass spectrometry (LC-MS/MS), which has been recommended by the Endocrine Society and has shown high sensitivity for steroid assay^11–13^, to explore the different profiles of steroid hormone and enzyme activity in the steroidogenic pathways of obese and nonobese PCOS patients. We hypothesized that obese and nonobese PCOS patients would have different steroid hormone profiles and therefore would present differences in the pathogenesis of PCOS.

## Results

### Anthropometric and biochemical characteristics

The baseline anthropometric and biochemical characteristics of the PCOS group and age- and BMI-matched controls are shown in Table 1. A total of 1044 women with PCOS (including 350 lean, 312 overweight and 382 obese patients), and 366 control women without PCOS (including 203 lean, 32 overweight and 131 obese controls) were analyzed in this study. No differences in age and BMI were found between any of the PCOS groups and their corresponding controls. The PCOS groups presented with increased liver enzymes, lipid profiles, plasma glucose and HOMA-IR compared with their corresponding controls. The obese PCOS patients showed higher liver enzymes, lipid profiles, plasma glucose and HOMA-IR compared with the lean PCOS patients (Table 1).

**Table 1:** Baseline anthropometric and biochemical characteristics.

	PCOS (n = 1044)			Control (n = 366)		
	BMI < 25 (n = 350)	25 ≤ BMI < 30 (n = 312)	BMI ≥ 30 (n = 382)	BMI < 25 (n = 203)	25 ≤ BMI < 30 (n = 32)	BMI ≥ 30 (n = 131)
Age (y)	26.0 (22.0, 30.0)	27.0 (22.0, 31.0)	26.0 (21.0–30.0)	26.0 (24.0, 32.0)	25.0 (20.0, 29.0)	25.0 (21.0, 30.0)
Weight (kg)	55.0 (51.0–60.0)^ab^	72.1 (68.5–77.1)^ac^	90.1 (84.0–100.6)^bc^	54.1 (51.4–58.0)	74.9 (69.3, 80.0)	91.0 (85.0–101.1)
BMI (kg/m2)	21.5 (19.6, 23.2)^ab^	27.6 (26.2, 29.0)^ac^	33.9 (31.9, 37.1)^bc^	21.0 (20.0, 22.2)	28.3 (25.1, 29.2)	33.7 (32.0, 37.4)
Waist (cm)	74.0 (68.0, 80.0)^abd^	91.0 (86.0, 95.0)^ac^	104.9 (99.0, 113.5)^bc^	73.0 (68.0, 79.0)^d^	91.25 (84.5, 95.0)	104.0 (98.0, 110.3)
Hip (cm)	92.0 (88.0, 95.0)^ab^	103.0 (100.0, 106.0)^ac^	112.3 (107.0, 119.6)^bc^	90.8 (88.0, 94.0)	104.5 (99.25, 109.0)	114.0 (107.5, 120.0)
WHR	0.81 (0.76, 0.86)^abd^	0.88 (0.84, 0.92)^ac^	0.93 (0.89, 0.99)^bc^	0.79 (0.74, 0.83)^d^	0.87 (0.83, 0.91)	0.91 (0.86, 0.96)
ALT(IU/L)	14.0 (11.0, 22.0)^abd^	23.0 (15.0, 47.0)^ace^	37.0 (23.0, 57.5)^bcf^	13.0 (11.0, 15.0)^d^	17.0 (12.5, 26.5)^e^	25.0 (19.0, 47.0)^f^
AST(IU/L)	17.0 (14.0, 21.8)^ab^	21.0 (16.0, 33.0)^ac^	25.0 (19.0, 35.0)^bd^	18.0 (16.0, 20.0)	18.0 (14.5, 21.0)^c^	21.0 (17.0, 28.0)^d^
ALP(IU/L)	51.0 (44.0, 62.0)^a^	58.0 (48.0, 75.0)	62.0 (55.0, 70.0)	45.0 (36.0, 52.0)^a^	54.0 (40.5, 67.0)	62.0 (52.0, 76.0)
GGT(IU/L)	13.0 (10.0, 18.0)^ab^	22.0 (15.0, 34.0)^acd^	27.0 (18.0, 42.0)^bce^	13.0 (10.8, 17.0)	15.0 (11.0, 17.0)^d^	20.0 (15.0, 32.0)^e^
TG(mmol/L)	0.88 (0.66, 1.26)^abc^	1.42 (1.04, 1.99)^ad^	1.48 (1.13, 2.11) ^b^	0.72 (0.58, 0.96)^c^	0.90 (0.72, 1.28)^d^	1.29 (1.03, 1.96)
TC(mmol/L)	4.33 (3.61, 4.95)^a^	4.54 (3.83, 5.28)	4.66 (4.02, 5.24)^a^	4.41 (3.88, 4.87)	4.66 (4.09, 5.15)	4.57 (4.04, 5.09)
HDL(mmol/L)	1.40 (1.07, 1.67)^ab^	1.10 (0.89, 1.34)^acd^	1.05 (0.93, 1.25)^bc^	1.51 (1.34, 1.66)	1.33 (1.17, 1.66)^d^	1.11 (0.96, 1.25)
LDL(mmol/L)	2.28 (1.79, 2.91)^abc^	2.69 (2.06, 3.20)^a^	2.89 (2.32, 3.35)^b^	2.52 (2.04, 2.93)^c^	2.72 (2.28, 3.17)	2.77 (2.37, 3.35)
FPG (mmol/L)	4.72 (4.50, 5.10)^ab^	5.00 (4.60, 5.40)^ac^	5.25 (4.81, 5.81)^bc^	4.83 (4.55, 5.00)	5.12 (4.80, 5.40)	5.20 (4.87, 5.29)
2hPG (mmol/L)	6.30 (5.38, 7.39)^abd^	7.30 (6.05, 9.04)^ac^	7.80 (6.54, 9.70)^bc^	5.42 (4.83, 6.24)^d^	6.80 (5.90, 8.22)	7.34 (6.18, 8.80)
HOMA-IR	1.91 (1.06, 3.45)^abd^	3,22 (1.95, 5.11)^ac^	5.28 (3.54, 7.71)^bce^	1.47 (1.11, 2.02)^d^	3.09 (2.16, 5.75)	4.34 (3.09, 6.20)^e^

Data are presented as median (25,75 percentile). FPG: fasting plasma glucose; 2hPG:2-hour plasma glucose. ^abcdef^The same superscripts indicate a statistically significant difference (P < 0.05) between pairs.

### Different steroid hormone profiles between the PCOS patients and controls in different BMI groups and among the three PCOS phenotypes

The levels of 14 steroid hormones measured with LC-MS/MS are presented in Table 2. When the lean, overweight and obese groups were analyzed separately according to BMI levels, the differences in steroid hormones were amplified in the lean PCOS patients versus the lean controls compared with the obese PCOS patients versus the obese controls. Briefly, both testosterone (T), androstenedione (AD2) and free androgen index (FAI) levels were significantly higher in the lean (all *P* < 0.001), overweight (*P* = 0.049, *P* = 0.005, *P* < 0.001) and obese (all *P* < 0.001) PCOS groups compared with the corresponding controls. Moreover, the lean PCOS patients showed increased DHEAS (*P* = 0.012), 17-hydropregnenolone (17-OHP5, *P* < 0.001), 17-hydroxyprogesterone (17-OHP, *P* = 0.001), 11-deoxycortisol (S, *P* < 0.001), cortisol (F, *P* = 0.019) and estrone (E1, *P* < 0.001) levels compared with the lean controls. The overweight PCOS group showed higher 17-OHP5(*P* = 0.037), S (*P* = 0.015) and F (*P* = 0.049), and lower 11-deoxycorticorsterone (DOC, *P* = 0.044) levels compared with the overweight controls, while the obese PCOS group only demonstrated lower E1 (*P* = 0.042) levels compared with the obese controls.

**Table 2:** Steroid hormone profiling measured with LC-MS/MS, and free androgen index (FAI) in PCOS patients and controls.

	PCOS (n = 1044)			Control (n = 366)		
	BMI < 25 (n = 350)	25 ≤ BMI < 30 (n = 312)	BMI ≥ 30 (n = 382)	BMI < 25 (n = 203)	25 ≤ BMI < 30 (n = 32)	BMI ≥ 30 (n = 131)
**Steroid hormones profiling (ng/ml)**						
DHEAS	1170.0 (798.5, 1595.0)^ab^	1040.0(735.0, 1470.0)	1080.0 (729.0, 1440.0)^a^	999.0 (728.5, 1392.5)^b^	918.0 (737.0, 1220.0)	930.5 (713.0, 1297.5)
17-hydropregnenolone	1.380 (0.723, 2.750)^ab^	1.150 (0.618, 2.150)^c^	1.095 (0.576, 2.023)^a^	0.889 (0.377, 1.750)^b^	0.816 (0.435, 1.620)^c^	1.180 (0.605, 2.710)
Aldosterone	0.038 (0.022, 0.066)	0.041 (0.023,0.064)	0.038 (0.024, 0.069)	0.037 (0.024, 0.059)	0.038 (0.028, 0.059)	0.037 (0.021, 0.064)
Corticosterone	1.770 (0.977, 2.990)	1.425 (0.856, 2.523)	1.515 (0.927, 2.983)	1.550 (0.960, 2.670)	1.580 (1.088, 2.090)	1.750 (0.953, 3.070)
Cortisol	88.80 (60.28, 124.00)^a^	82.40 (56.50, 114.00)^b^	85.49 (56.58, 118.66)	73.50 (53.10, 102.00)^a^	68.00 (55.43, 88.90)^b^	81.60 (55.80, 117.00)
Cortisone	29.50 (23.98, 36.85)	28.30 (22.24, 38.20)	30.19 (22.93, 39.45)	29.30 (23.00, 38.50)	31.30 (22.50, 39.33)	29.70 (21.50, 41.00)
Estrone	0.718 (0.457, 1.120)^ac^	0.767 (0.455, 1.280)^b^	0.551 (0.366, 0.841)^abd^	0.497 (0.320, 0.769)^c^	0.471 (0.310, 1.183)	0.712 (0.379, 1.100)^d^
Pregnenolone	10.600 (6.150, 20.600)	13.350 (6.788, 23.775)	12.100 (7.535, 22.550)	12.370 (7.385, 21.424)	12.450 (7.350, 18.975)	13.700 (8.860, 21.300)
Deoxycorticosterone	0.038 (0.025, 0.059)	0.035 (0.022, 0.050)^a^	0.033 (0.021, 0.055)	0.040 (0.028, 0.060)	0.041 (0.029, 0.054)^a^	0.036 (0.023, 0.065)
17-hydroprogesterone	0.331 (0.196, 0.744)^ac^	0.325 (0.188, 0.668)^b^	0.231 (0.130, 0.421)^ab^	0.270 (0.116, 0.710)^c^	0.273 (0.097, 0.772)	0.184 (0.074, 0.475)
11-deoxycortisol	0.337 (0.176, 0.589)^a^	0.284 (0.164, 0.497)^b^	0.300 (0.183, 0.493)	0.250 (0.150, 0.390)^a^	0.187 (0.094, 0.384)^b^	0.322 (0.143, 0.535)
Progesterone	0.277 (0.139, 0.984)^a^	0.262 (0.131, 0.825)^b^	0.154 (0.070, 0.690)^ab^	0.476 (0.121, 1.800)	0.213 (0.070, 3.315)	0.218 (0.070, 0.977)
Androstenedione	2.290 (1.683, 3.183)^a^	2.225 (1.670, 3,148)^b^	2.525 (1.780, 3.218)^c^	1.830 (1.360, 2.260)^a^	1.950 (1.518, 2.298)^b^	1.860 (1.430, 2.610)^c^
Testosterone	0.434 (0.310, 0.603)^b^	0.420 (0.291, 0.570)^ac^	0.479 (0.347, 0.661)^ad^	0.328 (0.260, 0.416)^b^	0.338 (0.261, 0.441)^c^	0.345 (0.248, 0.446)^d^
**Free androgen index (FAI)**						
FAI	1.37 (0.81, 2.19)^ade^	1.86 (1.16, 3.01)^bdf^	2.82 (1.75, 4.67)^cef^	0.55 (0.39, 0.76)^a^	0.77 (0.49, 1.09)^b^	1.77 (0.91, 2.86)^c^

Data are presented as median (25,75 percentile). ^abcdef^The same superscripts indicate a statistically significant difference (P < 0.05) between pairs.

When the three PCOS phenotypes and controls were compared (see Supplementary Table S1), T (all *P* < 0.001), AD2 (*P* = 0.002, *P* < 0.001, *P* < 0.001) and FAI (*P* = 0.003, *P* < 0.001, *P* < 0.001) were significantly higher in the three PCOS phenotype groups (CA + PCOm, HA + CA, HA + CA + PCOm) compared with the controls. Moreover, T (*P* < 0.001), AD2 (*P* < 0.001), DHEAS (*P* < 0.001), 17-OHP (*P* = 0.003), 17-OHP5 (*P* < 0.001), F(*P* = 0.020), S(*P* = 0.003) and FAI (*P* < 0.001) increased progressively in accordance with phenotype severity, with the highest values observed for the HA + CA + PCOm phenotype.

### Different steroid hormone profiles and enzyme activity among PCOS patients in different BMI groups

**Figure 1:**
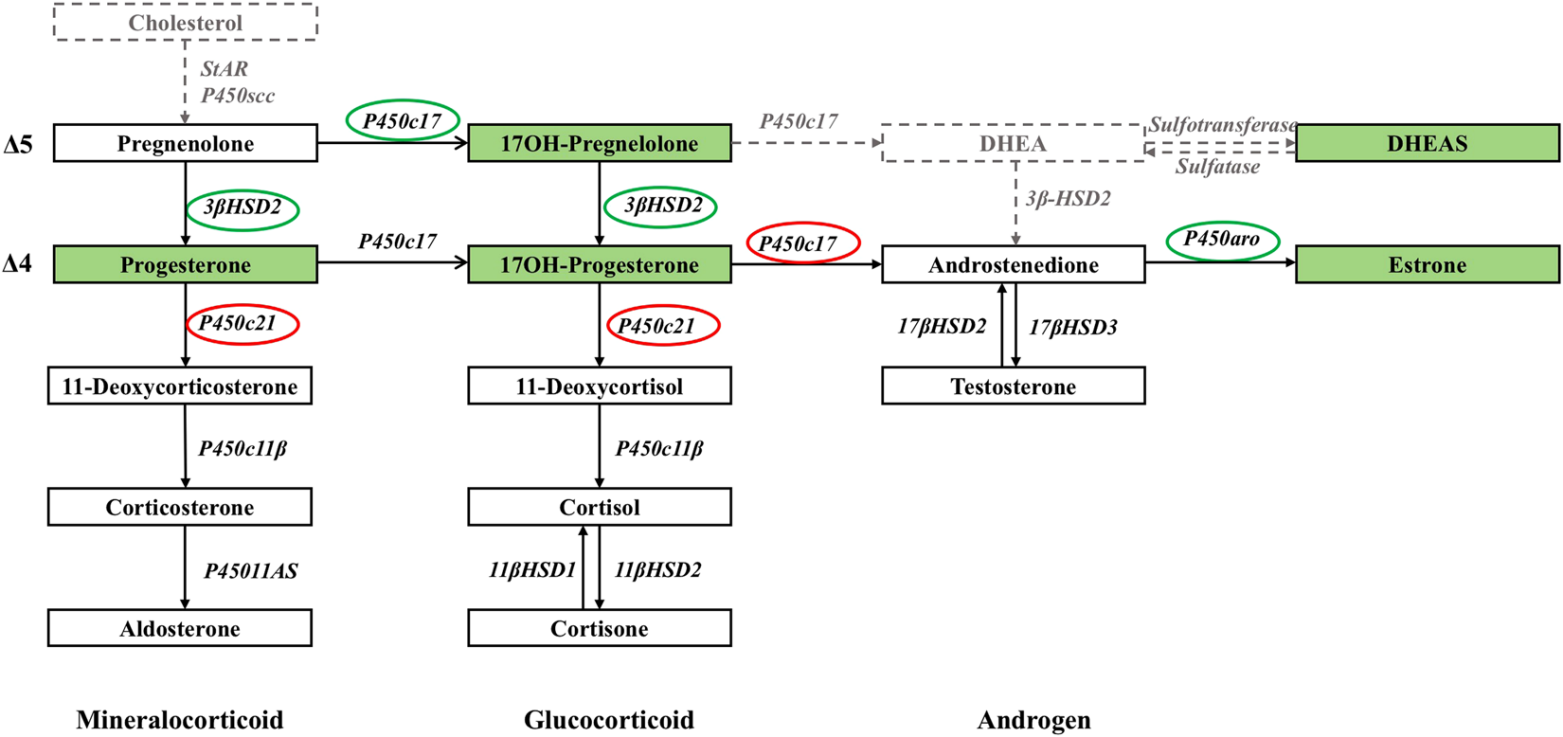
Schematic representation of the steroidogenic pathways including the differences between the lean and obese PCOS patients. The steroid hormones measured in this study are indicated by solid black lines, and those not measured in this study are indicated by broken gray lines. The steroid hormones that were higher in the lean PCOS patients than the obese PCOS patients are shown in the green box. The enzymes that showed higher activity levels in the lean PCOS patients are shown in the green circle, and those that had lower activity levels in the lean PCOS patients are shown in the red circle.

The differences in steroid hormone profiles among the PCOS patients in different BMI groups are explored in Table 2 and Fig. 1. Higher levels of DHEAS (*P* = 0.015), 17-OHP5 (*P* = 0.003), 17-OHP (*P* < 0.001), progesterone (P, *P* < 0.001) and E1 (*P* < 0.001), and lower FAI (*P* < 0.001) were observed in the lean PCOS group compared with the obese PCOS group. Similarly, higher levels of 17-OHP (*P* < 0.001), P (*P* = 0.002) and E1 (*P* < 0.001), and lower levels of T (*P* = 0.005) and FAI (*P* = 0.004) were found in the overweight PCOS group compared with the obese PCOS group, while only a lower FAI (*P* = 0.001) level was found in the lean PCOS group compared with the overweight PCOS group.

The enzyme activity in the steroidogenic pathway was further analyzed (Table 3 and Fig. 1). Compared with the obese PCOS group, the lean PCOS group showed increased activity of P450c17 (17-OHP5/ pregnenolone) (*P* < 0.001), P450aro (E1/AD2) (*P* < 0.001), and 3βHSD2 (P/ pregnenolone and 17-OHP/17-OHP5, both *P* < 0.001), and decreased activity of P450c17 (AD2/17-OHP) (*P* < 0.001), P450c21 (DOC/P) (*P* = 0.001, see Supplementary Fig. S2A), and P450c21 (S/17-OHP) (*P* < 0.001, see Supplementary Fig. S2B).

**Table 3:** Enzyme activity evaluation in PCOS patients and controls.

Enzyme	Product/precursor ratio	PCOS (n = 1044)			Control (n = 366)		
		BMI < 25 (n = 350)	25 ≤ BMI < 30 (n = 312)	BMI ≥ 30 (n = 382)	BMI < 25 (n = 203)	25 ≤ BMI < 30 (n = 32)	BMI ≥ 30 (n = 131)
P450c17	17-OHP5/P5	0.120 (0.057, 0.304)^abc^	0.093 (0.041, 0.204)^a^	0.088 (0.044, 0.198)^b^	0.082 (0.029, 0.192)^c^	0.096 (0.029, 0.162)	0.099 (0.046, 0.221)
P450c17	17-OHP/P	1.087 (0.370, 2.565)^a^	1.170 (0.406, 2.371)	1.302 (0.322, 3.153)	0.500 (0.136, 1.577)^a^	0.789 (0.203, 3.038)	0.997 (0.321, 2.143)
P450c17	AD2/17-OHP	6.999 (2.971, 11.750)^a^	7.747 (3.468, 12.580)^b^	11.296 (7.055, 17.476)^ab^	7.627 (2.773, 13.862)	5.684 (2.832, 17.910)	11.333 (3.473, 16.412)
P450c21	DOC/P	0.120 (0.043, 0.253)^a^	0.116 (0.046, 0.232)^b^	0.207 (0.050, 0.474)^ab^	0.067 (0.022, 0.311)	0.220 (0.022, 0.501)	0.154 (0.042, 0.443)
P450c21	S/17-OHP	0.883 (0.384, 2.154)^a^	0.773 (0.378, 1.824)^b^	1.362 (0.608, 2.795)^ab^	0.810 (0.330, 2.296)	0.888 (0.222, 2.554)	1.224 (0.503, 3.133)
P450c11β	B/DOC	46.98 (28.76, 74.44)^a^	44.73 (28.26, 71.36)	48.50 (28.05, 81.60)	39.00 (21.67, 69.33)^a^	33.88 (23.01, 67.74)	53.15 (24.55, 81.33)
P450c11β	F/S	242.5 (151.7, 409.5)^a^	274.4 (166.0, 397.8)	255.3 (170.9, 383.2)	293.2 (198.4, 427.6)^a^	295.9 (216.5, 465.7)	259.8 (178.0, 396.6)
P450aro	E1/AD2	0.320 (0.175, 0.563)^a^	0.322 (0.175, 0.623)^b^	0.219 (0.133, 0.391)^abc^	0.280 (0.172, 0.457)	0.263 (0.183, 0.516)	0.347 (0.191, 0.661)^c^
P450c11AS	Aldo/B	0.022 (0.010, 0.045)	0.027 (0.013, 0.053)	0.025 (0.012, 0.047)	0.023 (0.011, 0.041)	0.027 (0.017, 0.051)	0.021 (0.008, 0.040)
3βHSD2	P/P5	0.030 (0.011, 0.112)^a^	0.022 (0.008, 0.086)^b^	0.015 (0.005, 0.076)^ab^	0.036 (0.009, 0.152)	0.014 (0.005, 0.246)	0.018 (0.006, 0.089)
3βHSD2	17-OHP/17-OHP5	0.253 (0.113, 0.591)^ac^	0.294 (0.143, 0.583)^b^	0.194 (0.099, 0.442)^ab^	0.380 (0.155, 0.684)^c^	0.317 (0.111, 1.424)	0.204 (0.923, 0.404)
11βHSD2	C/F	0.355 (0.244, 0.490)^a^	0.377 (0.259, 0.506)^b^	0.382 (0.256, 0.563)	0.389 (0.318, 0.556)^a^	0.470 (0.345, 0.632)^b^	0.386 (0.257, 0.531)
17βHSD3	T/AD2	0.187 (0.153, 0.237)	0.184 (0.150, 0.221)	0.190 (0.157, 0.236)^a^	0.187 (0.162, 0.213)	0.184 (0.148, 0.233)	0.176 (0.140, 0.218)^a^

Data are presented as median (25,75 percentile). ^abcdef^The same superscripts indicate a statistically significant difference (P < 0.05) between pairs.

### Correlation of BMI with steroid hormones and enzyme activity

When a correlation analysis was conducted for the PCOS group, negative correlations were found between BMI and DHEAS (r = −0.110, *P* = 0.001), 17-OHP (r = −0.10, *P* < 0.001), P (r = −0.075, *P* = 0.023) and E1 (r = −0.104, *P* < 0.001) (see Supplementary Table. S2).

Regarding enzyme activity, negative correlations were found between BMI and P450c17 (17-OHP5/ pregnenolone) (r = −0.097, *P* = 0.004), P450aro (r = −0.162, *P* < 0.001), 3βHSD2 (17-OHP/17-OHP5) (r = −0.183, *P* < 0.001), and 3βHSD2 (P/P5) (r = −0.149, *P* < 0.001); positive correlations were found between BMI and P450c21 (DOC/P) (r = 0.157, *P* < 0.001), and P450c21 (S/17-OHP) (r = 0.157, *P* < 0.001) (see Supplementary Table S3).

### Different frequencies of single nucleotide polymorphism (SNP) among PCOS patients in different BMI groups

Since differences in P450c17, P450c21 3βHSD2, and P450aro were observed between the lean and obese PCOS patients (Fig. 1) and the former three enzymes mainly function in the adrenals, we further sequenced their encoding genes including CYP17A1, CYP21A2 and HSD3B2 in 216 PCOS patients with hyperandrogenism [defined as patients with increased T or free testosterone (FT) levels measured using immunoassay based on the clinical reference intervals].

In total, we found 25 variations in CYP21A2, 7 in CYP17A1 and 4 in HSD3B2 (see Supplementary Table S4). None of these mutations are pathogenic. When comparing the frequencies between the lean and obese PCOS groups, the lean PCOS patients demonstrated significantly higher frequencies of CYP21A2 c.552 C > G (p. D184E) compared with the obese PCOS patients (8.2% vs. 0%, *P* = 0.006; see Supplementary Fig. S3 and Table S5), while no significant differences were found in CYP17A1 and HSD3B2. The corresponding enzyme activity evaluations of the 216 PCOS patients are presented in Supplementary Table S6. The results were consistent with those of the larger group of 1044 PCOS patients.

## Discussion

The present study is the first to conduct a thorough comparison of steroid hormones between obese and nonobese PCOS patients. 14 steroid hormones were measured in 1044 PCOS patients and 366 controls using LC-MS/MS. Differences in steroid hormones were amplified in the lean PCOS patient versus the lean controls compared with the obese PCOS patients versus the obese controls. The lean and obese PCOS patients as well as all three PCOS phenotypes further presented with different steroid hormone profiles. The lean PCOS patients showed increased DHEAS, 17-OHP, 17-OHP5 and E1 levels compared with both the lean controls and the obese PCOS patients, while lower FAI were found in the lean PCOS patients compared with the obese PCOS patients. In addition, the lean PCOS patients showed increased activity of P450c17 (17-OHP5/P5), P450aro, and 3βHSD2, and decreased activity of P450c21. Furthermore, we identified a higher frequency of CYP21A2 (encoding P450c21) c.552 C > G (p. D184E) in the lean PCOS patients compared with the obese PCOS patients.

Steroid hormone measurements in PCOS patients have been previously reported in several studies^14–19^. Saito *et al*.^15^ measured the steroid hormones involved in androgen biosynthesis. However, the hormones in adrenal-specific pathways were not measured. Keefe *et al*.^14^ simultaneously measured 13 steroid hormones and presented a thorough picture of the steroid hormone profiles; however, the PCOS patients were not further analyzed according to BMI-related subgroups. Several studies have explored BMI-related differences in PCOS^16,17^; however, they mainly focused on the end-products of androgen, such as T, AD2 and DHEAS. The possible differences in intermediate steroid hormones between obese and nonobese PCOS patients, and the underlying mechanism were not fully described.

In the present study, we performed a comprehensive measurement of steroid hormones in the steroidogenic pathway in PCOS patients using LC-MS/MS. We found that T demonstrated an increased tendency in obese compared with nonobese PCOS patients, although no significant difference was observed. It has been reported that T levels cannot reflect hyperandrogenism precisely because 85% of total T is combined with SHBG in circulation with little biological activity^20^. Instead, FAI is considered to be a sensitive measurement that can accurately reflect the bioactive androgen levels^21,22^. Therefore, we further investigated the differences in FAI among the different BMI groups. The results showed that FAI increased progressively in the three BMI groups, with the highest value presenting in the obese PCOS patients. The correlation analysis in this study showed that FAI was positively correlated with BMI (r = 0.411, *P* < 0.001) and HOMA-IR (r = 0.378, *P* < 0.001). These results, in parallel with previous studies^23,24^, suggest that obesity-related insulin resistance may play a key role in the androgen excess of obese PCOS patients.

However, lean PCOS patients present less insulin resistance, which suggests that the pathogenesis in this group may differ from that of obese PCOS patients. In the present study, although FAI was decreased in the lean PCOS patients, DHEAS, the adrenal-specific androgen^7^, was increased in the lean PCOS patients compared with the lean controls and obese PCOS patients. This finding is consistent with previous observations suggesting higher DHEAS levels in PCOS patients with lower BMI^8–10^, and suggests that the adrenal androgen excess plays different roles in lean and obese PCOS patients. Moreover, we found that 17-OHP was increased in the lean PCOS patients compared with both the lean controls and the obese PCOS patients. Elevated 17-OHP levels are generally found in patients with specific adrenal disorders, such as congenital adrenal hyperplasia (CAH). Patients with CAH, especially non-classic congenital adrenal hyperplasia (NCAH), also demonstrated several clinical features, such as hyperandrogenism, menstrual irregulation and infertility, which are similar to those of PCOS patients^25^. A common procedure that distinguishes between NCAH and PCOS is the measurement of 17-OHP levels^26^. It has been commonly agreed that 17-OHP > 10 ng/mL indicates the existence of CAH with P450c21 deficiency, whereas values between 2 and 10 ng/mL require further differentiation between PCOS and adrenal disorders, especially NCAH^27^. The importance of 17-OHP evaluation in diagnostic studies has been emphasized by the American Association of Clinical Endocrinologists (AACE) and the Androgen Excess and PCOS Society (AES)^27^. In the present study, a higher level of 17-OHP was observed in the lean PCOS patients compared with the lean controls and obese PCOS patients, suggesting that the adrenal androgen excess might be partially involved in the pathogenesis of PCOS patients, especially those with a low BMI. This finding indicates the need for great caution when differentiation of PCOS patients from NCAH patients in this sub-group.

We also analyzed the enzyme activity in the steroidogenic pathway. We found that lean and obese PCOS patients demonstrated different enzyme activity for P450c17, P450c21, 3βHSD2, and P450aro; among these, P450c21 (catalyzing the 21-hydroxylation of P to DOC and 17-OHP to S) was reported to be found solely in the adrenals^28^. In this study, the enzyme activity of P450c21 decreased in the lean PCOS patients compared with the obese PCOS patients. To date, few studies have compared the differences in P450c21 activity between lean and obese PCOS patients, and the underlying mechanism remains unknown. It has been reported that P450c21 deficiency caused by pathogenic mutations of CYP21A2 was responsible for over 90% of CAH and leads to varying degrees of serum elevations of 17-OHP^29^. Therefore, to explain the discrepancies between lean and obese PCOS patients, gene polymorphisms analysis was further applied to explore the potential mechanism. We found that although no pathogenic mutation of CYP21A2 was observed, the frequency of CYP21A2 c.552 C > G (p. D184E) was significantly higher in the lean PCOS patients compared with the obese PCOS patients. This SNP of CYP21A2 might partially explain the differences in P450c21 enzyme activity and the discrepancies in upstream steroid hormone levels between the obese and lean PCOS patients. However, further basic molecular and cellular studies are needed to validate the current findings.

The decreased P450c21 activity in the lean PCOS group further confirmed our finding that the adrenal androgen excess played an important role in these patients, adding evidence to previous studies with DHEAS^9,10^. Several treatments are available for PCOS patients, including anti-androgen therapy and insulin-sensitizing agents^30^. PCOS therapies that target at improving insulin resistance are quite well established and are reported to reduce both insulin-enhanced ovarian androgen synthesis and the insulin-mediated inhibition of SHBG^31,32^. However, these therapies do not always obtain the desired effects in clinical practice^33–35^. Unluhizarci *et al*.^35^ reported that metformin didn’t improve P450c17 activity and had no effect on 17-OHP and AD2 responses to adrenocorticotropic hormone (ACTH). Vanky *et al*.^36^ found that in metformin-treated PCOS patients who didn’t experience relief from all symptoms, dexamethasone therapy achieved the greatest reductions in androgen levels in those with the highest adrenal androgen production. These results suggest that in addition to ovarian androgen excess and metabolic dysfunction, adrenal androgen excess cannot be underestimated in the pathogenesis of these patients. For a long time, researchers have been focusing on which sub-group of PCOS should be cautiously differentiated as having adrenal androgen excess. The present study shows that lean PCOS patients are likely to present with adrenal androgen excess, adding necessary evidence to support optimal treatment choices for PCOS patients with different pathogeneses.

The present study illustrated a comprehensive profile of steroid hormones and related enzyme activity in a relatively large sample of PCOS patients using the LC-MS/MS method. Differences between PCOS patients and controls, and between obese and non-obese PCOS patients were thoroughly explored, and gene polymorphisms analysis was further applied to explain the potential underlying mechanism. However, a limitation was that the gene sequencing was performed only for those with hyperandrogensim (n = 216). Nevertheless, the corresponding enzyme activity of 216 PCOS patients was further analyzed, and the results were consistent with those of 1044 PCOS patients. In this study, gene sequencing simply provides a possible explanation for the discrepancies in enzyme activity; however, further mechanism research and interventional trials are needed to validate our current findings.

In conclusion, we found that the differences in steroid hormones were amplified in lean PCOS patients versus lean controls compared with those of obese PCOS patients versus obese controls; furthermore, lean and obese PCOS patients presented with different steroid hormones profiles. Additionally, the study demonstrated for the first time that lean PCOS patients showed decreased activity of the enzyme P450c21, which functioned solely in the adrenals, compared with obese PCOS patients. The study suggested that the adrenal androgen excess played different roles in lean and obese PCOS patients, which represented as different enzyme activity in the steroidogenic pathway. Identifying the origin of androgen excess would help to determine the etiology and choose the optimal treatments for these patients.

## Methods

### Participants

From January 2006 to October 2016, 1346 patients with PCOS were screened at Shanghai Ruijin Hospital (n = 804) and Shanghai Renji Hospital (n = 542), both affiliated with Shanghai Jiaotong University School of Medicine (see Supplementary Fig. S1). PCOS was diagnosed according to the revised Criteria of Rotterdam^37^, based on the presence of two or more of the following: (1) chronic anovulation (CA): menstrual cycles at ≥35-day intervals or <10 bleeds per year, and polymenorrhea as ≤25 days; (2) hyperandrogenism (HA): total T or FT higher than reference intervals established in our laboratory or clinical manifestations of hyperandrogenism; (3) polycystic ovarian morphology (PCOm): ≥12 follicles (2–9 mm in diameter) in at least one ovary and/or ovarian volume >10 cm^3^. The PCOS population was categorized as three distinct phenotypes according to the combination of the classic criteria, including HA, CA, and PCOm^38^: (1) patients with CA and PCOm; (2) patients with HA and CA; (3) patients with HA, CA and PCOm (see Supplementary Table S1). Exclusion criteria included pregnancy; other related endocrine disorders, including congenital adrenal hyperplasia, thyroid dysfunction, Cushing syndrome and hyperprolactinemia; the use of medication used known to affect gonadal or adrenal function, energy metabolism or lipid metabolism within the 3 months before sample collection; and a history of ovarian surgery. Therefore, among the 1346 PCOS patients, 79 were excluded for having received related medical therapy within 3 months, 35 for a history of other endocrine disorders, 81 for a lack of serum samples, 62 for missing clinical data, and 45 for other reasons. Therefore, a total of 1044 PCOS patients were finally analyzed in this study.

In addition, 366 age- and BMI- matched control women without PCOS were enrolled from the database of healthy volunteers at Ruijin hospital (see Supplementary Fig. S1). The exclusion criteria for the control women included a diagnosis of PCOS, isolated anovulation, isolated hyperandrogensim, pregnancy, other related endocrine disorders, the use of medication known to affect gonadal or adrenal function, energy metabolism or lipid metabolism within the 3 months before sample collection, and a history of ovarian surgery.

This observational, cross-sectional study was approved by the Ethics Committee of Ruijin Hospital and Renjin Hospital, both affiliated with Shanghai Jiaotong University School of Medicine, and it complied with the Helsinki Declaration. Written informed consent was obtained from the PCOS patients and control women.

### Anthropometric measurements

A thorough medical history, including menstrual history, height, and waist and hip circumference were recorded for each patient and control woman. The BMI was calculated by dividing weight (kg) by squared height (m). PCOS patients and control women with a BMI lower than 25 kg/m^2^ were considered as lean; those with a BMI of 30 kg/m^2^ or higher were classified as obese, and those with a BMI between 25 kg/m^2^ and 30 kg/m^2^ were considered as overweight.

### Biochemical measurements

During the screening period, blood samples were drawn after an overnight fast of 10–12 h and each participant underwent a 75-g oral glucose tolerance test (OGTT). Liver enzymes (serum alanine

aminotransferase [ALT], aspartate aminotransferase [AST], alkaline phosphatase [ALP], g-glutamyl transferase [GGT] and a lipid profile, including triglycerides (TG), total cholesterol (TC), high-density lipoprotein (HDL) cholesterol, and low-density lipoprotein (LDL) cholesterol were measured using an autoanalyzer (Beckman CX-7 Biochemical Autoanalyzer, Brea, CA, USA). Plasma glucose was measured using an enzymatic method (Beckman CX-7 Biochemical Autoanalyzer, Brea, CA, USA). Serum insulin was measured by radioimmunoassay (Sangon Company, Shanghai, China). HOMA-IR was calculated as fasting insulin (FINS, uIU/ml) × fasting plasma glucose (FPG, mmol/L)/22.5.

Sex hormone measurements were all performed at Shanghai Institute of Endocrine and Metabolic Diseases as previously described^39^. Briefly, T was analyzed using chemiluminescence immunoassay (Abbott, Dundee, UK); FT was analyzed using radioimmunoassay (Beckman Coulter, Prague, Czech Republic). Westguard rules were followed for the quality control procedures. The intra- and inter-assay coefficients of variation (CV) for T were less than 4.9% and 8.0%, respectively. The intra- and inter-assay CV for FT were less than 6.2% and 9.7%, respectively. Other sex hormones including 17-hydroxyprogesterone (17-OHP) and androstenedione (AD2) were analyzed using radioimmunoassay (Beckman Coulter, Prague, Czech Republic); DHEAS and sex hormone-binding globulin (SHBG) were analyzed using chemiluminescence immunoassay (Abbott, Wiesbaden, Germany).

### LC-MS/MS assay of steroid hormones

After the enrollment of the PCOS patients and controls, we measured 14 steroid hormones in the steroidogenic pathway, including pregnenolone (P5), 17-hydropregnenolone (17-OHP5), progesterone (P), 17-OHP, AD2, estrone (E1), 11-deoxycorticorsterone (DOC), corticosterone (B), aldosterone (Aldo), 11-deoxycortisol (S), cortisol (F), cortisone (C), T and DHEAS, using an ABSciex triple quadrupole mass spectrometer 4500 equipped with an Eksigent ultraHPLC system (ABSciex, Ontario, Canada). The column used was a Kinetex 2.6 μm XB-C18 (2.1 mm × 50 mm; Phenomenex, Torrance, CA, USA) that maintained at 45 °C. The mobile phases were (A) aqueous formic acid solution (0.1%) with (B) methanolic formic acid solution (0.1%) at a flow rate of 0.5 ml/min, and the final injection volume of each sample was 20 μl. All acquisitions were performed in positive-ion mode. For sample preparation, P5, 17-OHP5, P, 17-OHP, AD2, E1, DOC, B, Aldo, S, F, C and T were extracted from 100 ul serum using 1 ml extraction buffer (50% N-hexane + 50% ethyl acetate + internal standard [Toronto Research Chemicals, Canada] at a final concentration of 0.5 ng/mL), followed by evaporation under constant nitrogen flow and re-dissolution with 100 ul methanol: deionized water (1:9, v/v). For protein precipitation and extraction of DHEAS, 400ul ZnSO4 1 mM: methanol (1:9, v/v) was added to 100 ul serum before configuration. Then, 100 ul supernatant was extracted and diluted with 100 ul deionized water. Quantitative analyses were performed in multiple reaction-monitoring (MRM) mode. Each steroid was identified by comparing retention time and two mass transitions with a deuterated reference compound. The intra- and inter-assay CVs of these assays were less than 10.0% and 14.5%, respectively. The free androgen index (FAI) was calculated as the total T (measured by LC-MS/MS) *100/SHBG.

### Enzymes activity evaluation in the steroidogenic pathway

Product-precursor ratios were used to evaluate enzymes activity in the steroidogenic pathway^14,40^. Five cytochrome P450 enzymes (P450c17, P450c21, P450c11β, P450c11AS and P450aro) and three hydroxysteroid dehydrogenases (3βHSD, 11βHSD and 17βHSD) were calculated (Fig. 1). P450c17 catalyzed both 17α-hydroxylase and 17,20-lyase activity. In the steroidogenic Δ5 pathway, P450c17 was evaluated using the ratio of 17-OHP5/P5 (17α-hydroxylase). In the Δ 4 pathway, P450c17 was calculated using the ratios of 17-OHP/P (17α-hydroxylase) and AD2/17-OHP (17,20lyse, which is nearly negligible in the Δ4 pathway^28^). In the mineralocorticoid synthesis pathway, P450c21 was calculated using the ratio of DOC/P; P450C11β was calculated using the ratio of B/DOC; and P450c11AS was calculated using the ratio of Aldo/B, respectively. In the glucocorticoid synthesis pathway, P450c21 was calculated using the ratio of S/17-OHP; P450C11β was calculated using the ratio of F/S; and 11βHSD2 was calculated using the ratio of cortisone/F, respectively. In the androgen synthesis pathway, 17βHSD3 was calculated using the ratio of T/AD2. In the conversion from Δ5 steroids to Δ4 steroids, 3βHSD2 was calculated using the ratios of P/P5 and 17-OHP/17-OHP5, respectively. In the conversion from AD2 to E1, P450aro was calculated using the ratio of E1/AD2.

### Genetic variation in the steroidogenic pathway

Since differences in the three enzymes (P450c17, 3βHSD2 and P450c21) that mainly function in the adrenals were observed between obese and nonobese PCOS patients, sanger sequencing of CYP17A1 (encoding P450c17), HSD3B2 (encoding 3βHSD2) and CYP21A2 (encoding P450c21) was performed in 216 PCOS patients with hyperandrogenism (defined as patients with increased T or FT levels measured using immunoassay based on the clinical reference intervals) with a 3730xl DNA Analyzer (Applied Biosystems, Foster City, CA, USA).

### Statistical analysis

Statistical analysis was performed with SPSS (version 20.0 SPSS, Chicago, IL, USA). Continuous variables were summarized as medians (interquartile range), and categorical variables were summarized as proportions (categorical). Logarithmic transformation was implemented for variables that were not normally distributed. The independent-samples t-test was used for comparisons between two groups. One-way ANOVA and ANCOVA tests were used for multiple comparisons among different groups. Correlation testing was performed using Pearson’s correlation coefficient or Spearman’s test as appropriate. Categorical variables were analyzed using the chi-square test or Fisher exact test as appropriate. *P* < 0.05 was considered significant.

## Electronic supplementary material

Supplementary information

## References

[1] Lizneva D (2016). Criteria, prevalence, and phenotypes of polycystic ovary syndrome. Fertility and sterility.

[2] Randeva HS (2012). Cardiometabolic aspects of the polycystic ovary syndrome. Endocrine reviews.

[3] Azziz R (2016). Polycystic ovary syndrome. *Nature reviews*. Disease primers.

[4] Dumesic DA (2015). Scientific Statement on the Diagnostic Criteria, Epidemiology, Pathophysiology, and Molecular Genetics of Polycystic Ovary Syndrome. Endocrine reviews.

[5] Azziz R (2004). The prevalence and features of the polycystic ovary syndrome in an unselected population. The Journal of clinical endocrinology and metabolism.

[6] Yildiz BO, Azziz R (2007). The adrenal and p*o*lycystic ovary syndrome. Reviews in endocrine & metabolic disorders.

[7] Longcope C (1986). Adrenal and gonadal androgen secretion in normal females. Clinics in endocrinology and metabolism.

[8] Moran C, Knochenhauer E, Boots LR, Azziz R (1999). Adrenal androgen excess in hyperandrogenism: relation to age and body mass. Fertility and sterility.

[9] Kumar A, Woods KS, Bartolucci AA, Azziz R (2005). Prevalence of adrenal androgen excess in patients with the polycystic ovary syndrome (PCOS). Clinical endocrinology.

[10] Moran, C., Arriaga, M., Arechavaleta-Velasco, F. & Moran, S. Adrenal androgen excess and body mass index in polycystic ovary syndrome. *The Journal of clinical endocrinology and metabolism*, jc00009999, 10.1210/jc.0000-9999 (2015). 10.1210/jc.2014-2569 25514100

[11] Rosner W, Auchus RJ, Azziz R, Sluss PM, Raff H (2007). Position statement: Utility, limitations, and pitfalls in measuring testosterone: an Endocrine Society position statement. The Journal of clinical endocrinology and metabolism.

[12] Azziz R (2006). Positions statement: criteria for defining polycystic ovary syndrome as a predominantly hyperandrogenic syndrome: an Androgen Excess Society guideline. The Journal of clinical endocrinology and metabolism.

[13] Wierman ME (2006). Androgen therapy in women: an Endocrine Society Clinical Practice guideline. The Journal of clinical endocrinology and metabolism.

[14] Keefe CC (2014). Simultaneous measurement of thirteen steroid hormones in women with polycystic ovary syndrome and control women using liquid chromatography-tandem mass spectrometry. PloS one.

[15] Saito K (2016). Steroidogenic pathways involved in androgen biosynthesis in eumenorrheic women and patients with polycystic ovary syndrome. The Journal of steroid biochemistry and molecular biology.

[16] Kiddy DS (1990). Differences in clinical and endocrine features between obese and non-obese subjects with polycystic ovary syndrome: an analysis of 263 consecutive cases. Clinical endocrinology.

[17] Moran C (2008). Obesity differentially affects serum levels of androstenedione and testosterone in polycystic ovary syndrome. Fertility and sterility.

[18] Yasmin E, Balen AH, Barth JH (2013). The association of body mass index and biochemical hyperandrogenaemia in women with and without polycystic ovary syndrome. European journal of obstetrics, gynecology, and reproductive biology.

[19] Handelsman DJ, Teede HJ, Desai R, Norman RJ, Moran LJ (2017). Performance of mass spectrometry steroid profiling for diagnosis of polycystic ovary syndrome. Human reproduction (Oxford, England).

[20] Jayagopal V, Kilpatrick ES, Jennings PE, Hepburn DA, Atkin SL (2003). The biological variation of testosterone and sex hormone-binding globulin (SHBG) in polycystic ovarian syndrome: implications for SHBG as a surrogate marker of insulin resistance. The Journal of clinical endocrinology and metabolism.

[21] Pasquali R (2016). Defining Hyperandrogenism in Women With Polycystic Ovary Syndrome: A Challenging Perspective. The Journal of clinical endocrinology and metabolism.

[22] Vermeulen A, Verdonck L, Kaufman JM (1999). A critical evaluation of simple methods for the estimation of free testosterone in serum. The Journal of clinical endocrinology and metabolism.

[23] Li H (2016). Free androgen index and Irisin in polycystic ovary syndrome. Journal of endocrinological investigation.

[24] Al-Jefout M, Alnawaiseh N, Al-Qtaitat A (2017). Insulin resistance and obesity among infertile women with different polycystic ovary syndrome phenotypes. Scientific reports.

[25] Pall M, Azziz R, Beires J, Pignatelli D (2010). The phenotype of hirsute women: a comparison of polycystic ovary syndrome and 21-hydroxylase-deficient nonclassic adrenal hyperplasia. Fertility and sterility.

[26] Moran C, Azziz R (2003). 21-hydroxylase-deficient nonclassic adrenal hyperplasia: the great pretender. Seminars in reproductive medicine.

[27] Goodman NF (2015). American Association Of Clinical Endocrinologists, American College Of Endocrinology, And Androgen Excess And Pcos Society Disease State Clinical Review: Guide To The Best Practices In The Evaluation And Treatment Of Polycystic Ovary Syndrome–Part 1. Endocrine practice: official journal of the American College of Endocrinology and the American Association of Clinical Endocrinologists.

[28] Miller WL, Auchus RJ (2011). The molecular biology, biochemistry, and physiology of human steroidogenesis and its disorders. Endocrine reviews.

[29] Speiser PW, White PC (2003). Congenital adrenal hyperplasia. The New England journal of medicine.

[30] Legro RS (2013). Diagnosis and treatment of polycystic ovary syndrome: an Endocrine Society clinical practice guideline. The Journal of clinical endocrinology and metabolism.

[31] Moghetti P (2000). Metformin effects on clinical features, endocrine and metabolic profiles, and insulin sensitivity in polycystic ovary syndrome: a randomized, double-blind, placebo-controlled 6-month trial, followed by open, long-term clinical evaluation. The Journal of clinical endocrinology and metabolism.

[32] Pasquali R (2000). Effect of long-term treatment with metformin added to hypocaloric diet on body composition, fat distribution, and androgen and insulin levels in abdominally obese women with and without the polycystic ovary syndrome. The Journal of clinical endocrinology and metabolism.

[33] Harborne L, Fleming R, Lyall H, Norman J, Sattar N (2003). Descriptive review of the evidence for the use of metformin in polycystic ovary syndrome. Lancet (London, England).

[34] Mathur R, Alexander CJ, Yano J, Trivax B, Azziz R (2008). Use of metformin in polycystic ovary syndrome. American journal of obstetrics and gynecology.

[35] Unluhizarci K, Kelestimur F, Sahin Y, Bayram F (1999). The treatment of insulin resistance does not improve adrenal cytochrome P450c17alpha enzyme dysregulation in polycystic ovary syndrome. European journal of endocrinology/European Federation of Endocrine Societies.

[36] Vanky E, Salvesen KA, Carlsen SM (2004). Six-month treatment with low-dose dexamethasone further reduces androgen levels in PCOS women treated with diet and lifestyle advice, and metformin. Human reproduction (Oxford, England).

[37] Revised 2003 consensus on diagnostic criteria and long-term health risks related to polycystic ovary syndrome (PCOS). *Human reproduction (Oxford, England)* **19**, 41–47 (2004). 10.1093/humrep/deh098 14688154

[38] Health, N. I. o. Evidence-based methodology workshop on polycystic ovary syndrome, December 3–5, 2012.Executive summary. Available at: https://prevention.nih.gov/docs/programs/pcos/FinalReport.pdf. Accessed March 1, 2016).

[39] Liang P (2017). Prevalence of polycystic ovary syndrome in Chinese obese women of reproductive age with or without metabolic syndrome. Fertility and sterility.

[40] Naessen T (2010). Steroid profiles in ovarian follicular fluid in women with and without polycystic ovary syndrome, analyzed by liquid chromatography-tandem mass spectrometry. Fertility and sterility.

