# Supplementary material for: Steroid hormone profiling in obese and nonobese women with polycystic ovary syndrome
Source: Sci Rep. 2017 Oct 26;7:14156. doi: 10.1038/s41598-017-14534-2 (PMC5658378; doi:10.1038/s41598-017-14534-2)
Supplement: Supplementary file 1 — Supplementary information [file 41598_2017_14534_MOESM1_ESM.pdf]

Steroid hormone profiling in obese and nonobese women with polycystic ovary syndrome

Yuying Deng[a#], Yifei Zhang[a#], Shengxian Li[b#], Wenzhong Zhou[a], Lei Ye[a], Lihua Wang[b], Tao Tao[b], Junjie Gu[a], Zuwei Yang[a], Dandan Zhao[a], Weiqiong Gu[a], Jie Hong[a], Guang Ning[a], Wei Liu[b¶], Weiqing Wang[a¶]

[#] Yuying Deng, Yifei Zhang and Shengxian Li contributed equally to this work.

¶ Corresponding author: Weiqing Wang and Wei Liu.

Supplementary Figure. S1. Flow diagram.

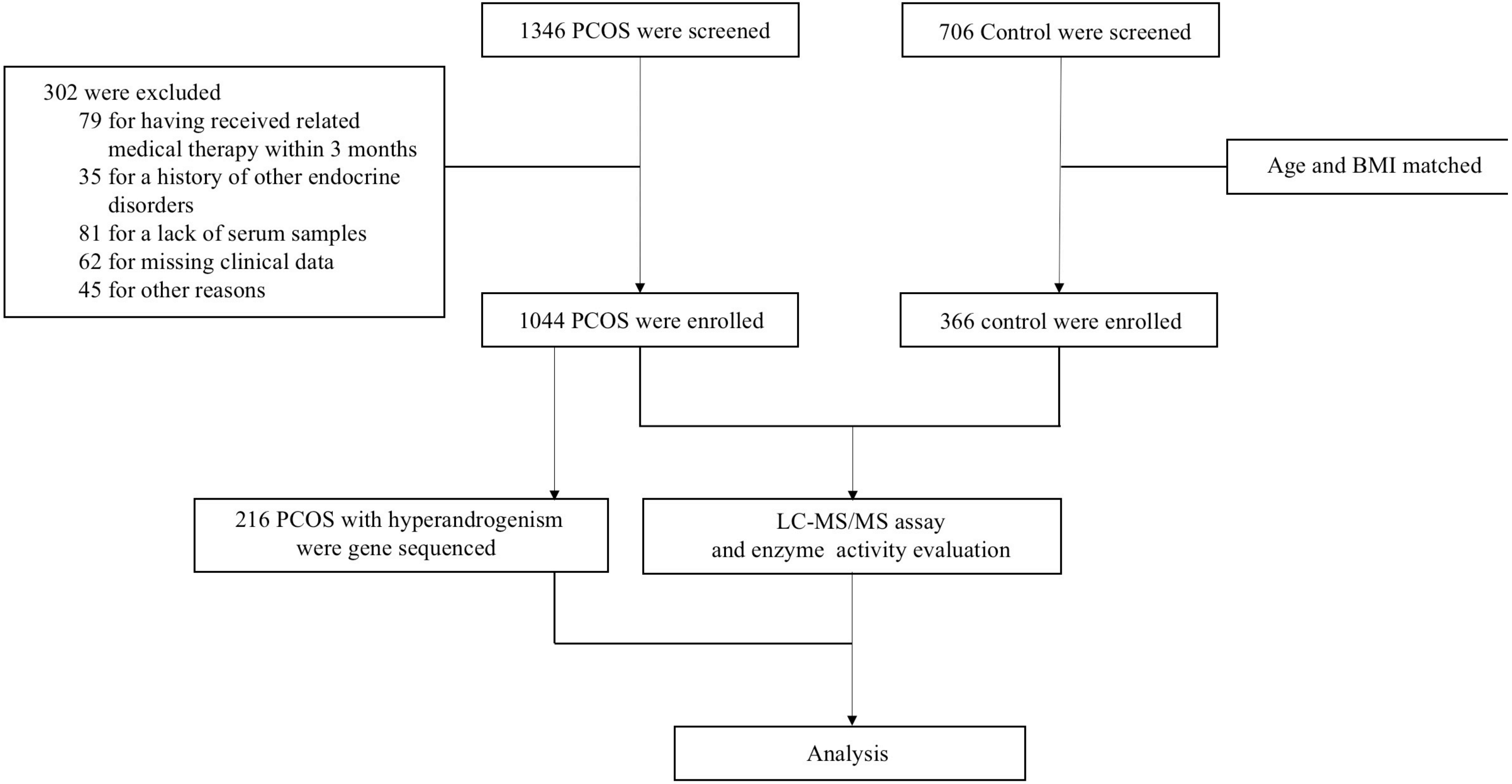

Supplementary Figure. S2. Enzymes activity of P450c21 in PCOS groups classified by BMI.

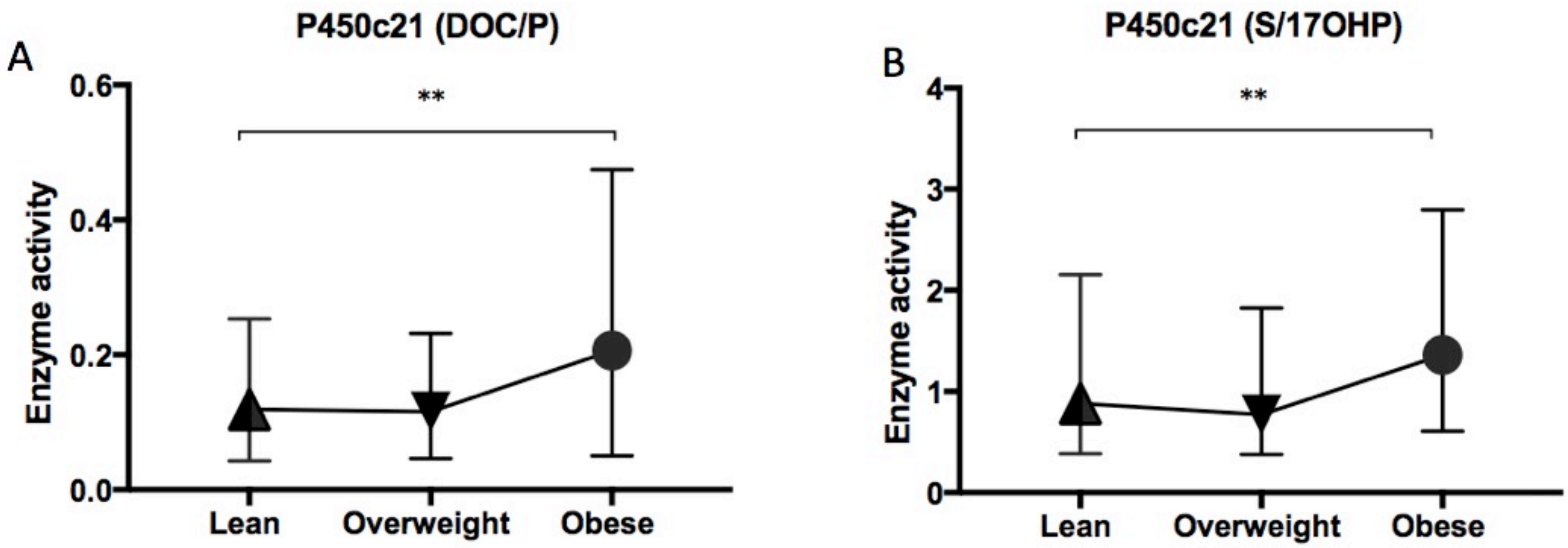

(A) The enzyme activity of P450c21 (DOC/P); (B) The enzyme activity of P450c21 (S/17-OHP).

Supplementary Fig. S3. Frequencies of CYP21A2 c. 552C>G (p. D184E) in 216 PCOS patients with hyperandrogenism classified by BMI.

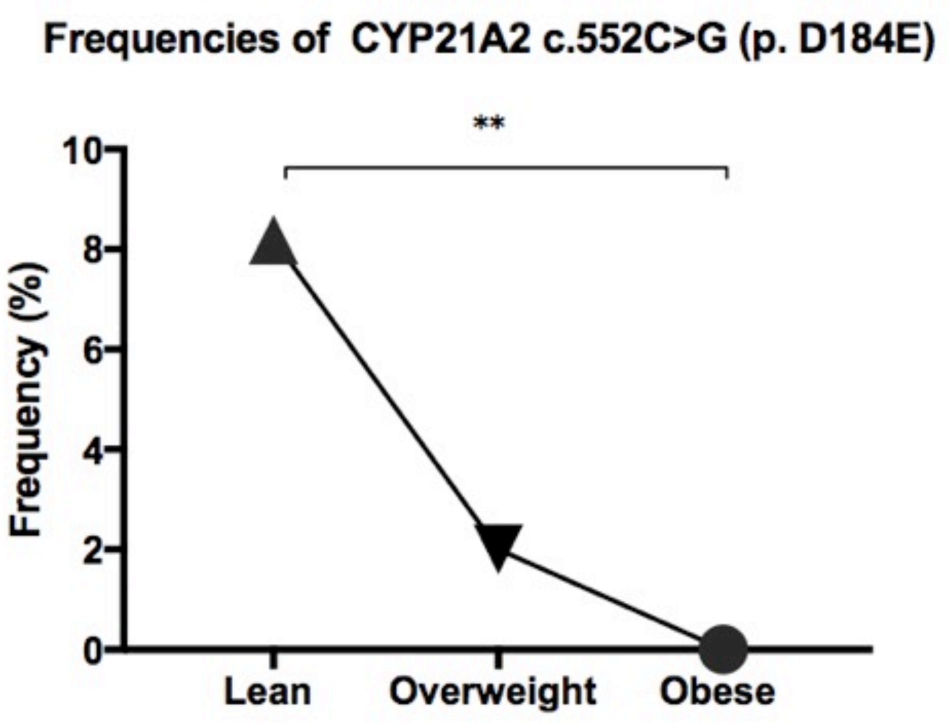

		PCOS Phenotypes			P for trend in PCOS
	Control (n=366)	CA + PCOm (n=418)	HA+CA (n=392)	HA+ CA+ PCOm (n=234)	
Age (y)	26.0 (23.0, 32.0)	27.0 (23.0, 32.0)	25.0 (20.0, 29.0) [a]	26.0 (21.0, 29.0)	0.126 [b]
BMI (kg/m2)	23.23 (20.74, 32.42)	27.93 (23.05, 32.23) [a]	30.41 (25.10, 34.72) [a]	27.04 (23.03, 31.09) [a]	0.073 [b]
DHEAS (ng/ml)	989.0 (728.0, 1365.0)	991.5 (678.5, 1400.0)	1110.0(755.0, 1500.0)	1210.0 (898.0, 1650.0) [a]	<0.001[c]
17-hydropregnenolone (ng/ml)	0.962 (0.450, 2.015)	1.050 (0.575, 1.983)	1.220 (0.680, 2.280) [a]	1.420 (0.823, 2.980) [a]	<0.001[c]
Aldosterone (ng/ml)	0.037 (0.022, 0.061)	0.038 (0.020, 0.062)	0.041 (0.023,0.064)	0.037 (0.023, 0.064)	0.124 [c]
Corticosterone (ng/ml)	1.620 (0.965, 2.723)	1.400 (0.843, 2.480) [a]	1.790 (1.013, 3.256)	1.705 (0.968, 3.090)	0.001 [c]
Cortisol (ng/ml)	73.95 (54.73, 105.00)	77.30 (53.88, 108.03)	88.35 (58.18, 121.00)	97.10 (67.80, 135.00) [a]	0.020 [c]
Cortisone (ng/ml)	29.50 (22.60, 39.60)	26.70 (21.56, 32.60)	32.35 (24.73, 42.03)	30.00 (24.50, 40.49)	<0.001[c]
Estrone (ng/ml)	0.536 (0.338, 0.915)	0.632 (0.384, 1.063) [a]	0.650 (0.415, 0.996)	0.746 (0.451, 1.300) [a]	0.057[c]
Pregnenolone (ng/ml)	13.10 (7.71, 21.10)	11.04 (6.33, 20.55)	14.07 (7.69, 23.50)	11.50 (6.58, 24.83)	0.070[c]
Deoxycorticosterone (ng/ml)	0.040 (0.025, 0.060)	0.030 (0.020, 0.047) [a]	0.039 (0.025, 0.063)	0.037 (0.025, 0.058)	0.015[c]
17-hydroprogesterone (ng/ml)	0.225 (0.104, 0.674)	0.218 (0.132, 0.530)	0.297 (0.161, 0.539)	0.439 (0.261, 0.826) [a]	0.003[c]
11-deoxycortisol (ng/ml)	0.263 (0.143, 0.432)	0.288 (0.165, 0.440)	0.330 (0.178, 0.600) [a]	0.362 (0.198, 0.596) [a]	0.003[c]
Progesterone (ng/ml)	0.304 (0.090, 1.523)	0.250 (0.114, 0.941)	0.179 (0.083, 0.665) [a]	0.299 (0.132, 1.163)	0.196[c]
Androstenedione (ng/ml)	1.858 (1.410, 2.343)	1.720 (1.350, 2.115) [a]	2.880 (2.300, 3.810) [a]	2.950 (2.298, 3.760) [a]	<0.001[c]
Testosterone (ng/ml)	0.332 (0.257, 0.426)	0.306 (0.247, 0.369) [a]	0.573 (0.474, 0.730) [a]	0.575 (0.474, 0.736) [a]	<0.001[c]
FAI	0.66 (0.45, 1.20)	1.42 (0.80, 2.10) [a]	2.30 (1.42, 3.63) [a]	2.77 (1.62, 4.52) [a]	<0.001[c]

Supplementary Table S1. Steroid hormone profiling measured with LC-MS/MS in the three PCOS phenotype groups and controls.

Data are presented as median (25,75 percentile). [a] P<0.05 vs controls; [C]ANVOVA for trend with age and BMI as covariate.

**Steroid hormones**	**r**	**P value**
DHEAS	-0.110	**0.001**
17OH-progesterone	-0.190	**<0.001**
Progesterone	-0.075	**0.023**
Estrone	-0.104	**0.002**
17OH-pregnenolone	-0.038	0.254
Aldosterone	0.055	0.110
Corticosterone	-0.013	0.694
Cortisol	-0.037	0.263
Cortisone	0.059	0.078
Pregnenolone	0.048	0.153
Androstenedione	0.014	0.671
11-deoxycortisol	-0.063	0.055
Deoxycorticosterone	-0.051	0.121
Testosterone	0.035	0.291

Supplementary Table S2. Correlation of BMI with steroid hormones in the PCOS groups.

**Enzyme**	**Product/precursor ratio**	**r**	**P value**
P450c17	17OHP5/P5	-0.097	**0.004**
P450c17	A/17OHP	0.014	0.673
P450c17	17OHP/P	0.034	0.309
P450c21	11DOC/P	0.157	**0.000**
P450c21	S/17OHP	0.157	**0.000**
P450c11β	F/S	0.040	0.223
P450c11β	B/DOC	0.023	0.497
P450aro	E1/AD2	-0.162	**0.000**
P450c11AS	Aldo/B	0.043	0.214
3βHSD2	17OHP/17OHP5	-0.183	**0.000**
3βHSD2	P/P5	-0.149	**0.000**
11βHSD2	Cortisone/F	0.055	0.102
17βHSD3	T/AD2	0.013	0.691

Supplementary Table S3. Correlation of BMI with enzymes activity in the PCOS groups.

Gene	Enzyme	Product/precursor ratio	Polymorphism sites
CYP17A1	P450c17	17OHP5/P5<br>17-OHP/P<br>A/17-OHP	c.195G>T, c.138C>T, c.152154delAAG, c.2325G>T, c.1098T>G, c.414C>A, c.32C>T.
CYP21A2	P450c21	DOC/P<br>S/17OHP	c.118C>T, c.138C>A, c.1481G>A, c.1473G>A, c.806G>C, c.747C>G, c.371C>T, c.308G>A, c.1125C>T, c.1618delCTG, c.12717G>A, c.933G>A, c.325326delTGinsCA, c.950G>A, c.79A>G, c.327C>G, c.332339delGAGACTAC, c.342C>T, c.518T>A, c.12716C>T, c.955C>T, c.45C>T, c.1456A>G, c.552C>G, c.844G>T.
HSD3B2	3βHSD2	P/P5<br>17OHP/17OHP5	c.924C>T, c.500C>T, c.465G>A, c.549G>A.

Supplementary Table S4. Single nucleotide polymorphisms (SNPs) in the steroidogenic pathway in 216 PCOS patients with hyperandrogenism.

PCOS group	CYP21A2 c. 552C>G (p. D184E)		Total
	With mutation	Without mutation	
BMI<25	5 (8.2%) [a]	56 (91.8%)	61
BMI 25-30	1 (2.0%)	48 (98.0%)	49
BMI ≥30	0 (0.0%) [a]	106 (100%)	106
Total	6 (2.8%)	210 (97.2%)	216

Supplementary Table S5. CYP21A2 c. 552C>G (p. D184E) frequencies in 216 obese and nonobese PCOS patients with hyperandrogenism.

Data are presented as number (%). [a] The same superscripts indicate a statistically significant difference ($P<0.05$) between pairs.

	BMI<25 (n=61)	25≤BMI <30 (n=49)	BMI≥30 (n=106)
CYP17A1			
P450c17 (17OHP5/P5)	0.262 (0.085, 0.663) [a]	0.147 (0.056, 0.389)	0.096 (0.054, 0.200) [a]
P450c17 (17OHP/P)	1.943 (0.366, 3.420)	0.719 (0.191, 2.108)	0.957 (0.183, 3.682)
P450c17 (AD2/17OHP)	6.950 (2.438, 10.924) [a]	9.074 (4.759, 13.546) [b]	12.000 (7.904, 16.038) [ab]
HSD3B2			
3βHSD2 (P/P5)	0.047 (0.018, 0.260)	0.045 (0.008, 0.364)	0.016 (0.006, 0.255)
3βHSD2 (17OHP/17OHP5)	0.241 (0.151, 0.633)	0.235 (0.124, 0.574)	0.187 (0.109, 0.467)
CYP21A2			
P450c21 (DOC/P)	0.138 (0.040, 0.263)	0.073 (0.015, 0.244)	0.147 (0.020, 0.416)
P450c21 (S/17OHP)	0.865 (0.301, 1.820)	0.772 (0.382, 2.342)	1.274 (0.567, 2.321)

Supplementary Table S6. Enzyme activity evaluation in 216 PCOS patients with hyperandrogenism.

Data are presented as median (25,75 percentile). [abcdef] The same superscripts indicate a statistically significant difference (P<0.05) between pairs.
